# Comparative performance of pre- and post-endoscopic risk scores for outcome prediction in endoscopy-confirmed upper gastrointestinal bleeding in the emergency department: A retrospective single-center study

**DOI:** 10.1097/MD.0000000000051268

**Published:** 2026-10-23

**Authors:** Emre Oner, Niyazi Ilhan, Yasar Catal, Sinan Genc, Ayca Koca, Muge Gunalp

**Affiliations:** a Department of Emergency Medicine, Ankara University School of Medicine, Ankara, Turkey; b Department of Emergency Medicine, Kayseri City Hospital, Kayseri, Turkey.

**Keywords:** AIMS65, Baylor score, emergency department, Glasgow-Blatchford score, Rockall score, upper gastrointestinal bleeding

## Abstract

Upper gastrointestinal bleeding (UGIB) requires early risk stratification, but commonly used scores were developed for different clinical moments and endpoints. We compared albumin, international normalized ratio, mental status, systolic blood pressure, age ≥65 years (AIMS65), Glasgow-Blatchford score (GBS), pre-Baylor, full Baylor, pre-Rockall, and full Rockall for the prediction of 30-day mortality, red-cell transfusion, endoscopic therapy, and rebleeding in an endoscopy-confirmed emergency department cohort. We retrospectively evaluated consecutive adults with suspected UGIB who underwent endoscopy and had an upper gastrointestinal bleeding source confirmed in the emergency department pathway between January 1, 2016 and 1 June 1, 2019. Thirty-day mortality was ascertained from hospital records and national death records. Rebleeding was defined as recurrent hematemesis and/or melena, or hemodynamic instability with rebleeding confirmed on repeat endoscopy, as documented in the available follow-up records. Score discrimination was assessed with receiver operating characteristic analysis. The analytic cohort comprised 347 patients (mean age 65.54 ± 15.82 years; 62.6% male). Index-hospital disposition included direct discharge after endoscopy in 27 patients (7.8%), discharge after hospitalization in 282 (81.3%), and in-hospital death in 38 (11.0%); the separate 30-day mortality endpoint used for receiver operating characteristic analysis comprised 46 deaths (13.3%). Blood transfusion occurred in 205 patients (59.1%), endoscopic therapy in 29 (8.4%), and rebleeding in 71 (20.5%). AIMS65 showed the highest area under the curve (AUC) for 30-day mortality (0.724, 95% confidence interval [CI] 0.647–0.802). GBS showed the highest AUC for transfusion (0.784, 95% CI 0.734–0.834) and rebleeding (0.703, 95% CI 0.639–0.767). For endoscopic therapy, only GBS reached nominal statistical significance, but discrimination was limited (AUC 0.604, 95% CI 0.551–0.656). In this selected cohort of endoscopy-confirmed ED patients with UGIB, score performance was outcome-specific rather than uniform across all endpoints. AIMS65 showed the highest mortality discrimination, whereas GBS showed the highest discrimination for transfusion and rebleeding. Endoscopic therapy prediction remained weak. These scores should be interpreted as structured adjuncts to clinical and endoscopic assessment, not as standalone decision rules.

## 1. Introduction

Upper gastrointestinal bleeding (UGIB) remains a common medical emergency associated with substantial morbidity, resource use, and short-term mortality. Contemporary guidelines emphasize early risk assessment to distinguish very-low-risk patients suitable for outpatient management from those who require urgent endoscopy, transfusion support, or monitored inpatient care.^[1,2]^

The Glasgow-Blatchford score (GBS) was derived as a pre-endoscopic tool to predict the need for treatment or hospital-based intervention and has remained the most widely used score for identifying low-risk patients, particularly at thresholds of 0 to 1 or 0 to 2 in appropriately selected pathways.^[3–5]^ By contrast, the Rockall and Baylor systems were constructed around clinical severity plus endoscopic findings, making them conceptually more relevant to post-endoscopic mortality or rebleeding assessment than to undifferentiated emergency department (ED) triage.^[6–8]^ Albumin, international normalized ratio, mental status, systolic blood pressure, age ≥65 years (AIMS65) was later proposed as a simple admission score aimed primarily at mortality and resource use.^[9]^

Comparative studies have consistently shown that score performance is endpoint-dependent. In multicenter and prospective cohorts, GBS has generally performed well for intervention-related outcomes and low-risk discharge, whereas AIMS65, full Rockall, or newer mortality-oriented models have at times shown better discrimination for death-related outcomes.^[10–17]^ More recent comparative work and evidence syntheses continue to show that no single score performs optimally across all clinical decisions or all time points of care.^[18–22]^

Recent emergency-unit comparisons have generally evaluated fewer score sets, different endpoint mixes, or hospitalization/high-risk composites rather than the combination of mortality, transfusion, rebleeding, and endoscopic treatment need.^[19,20]^ Whether both pre- and post-endoscopic models retain distinct, outcome-specific value when applied within an ED cohort selected for endoscopic confirmation therefore remains insufficiently defined. We compared AIMS65, GBS, pre-Baylor, full Baylor, pre-Rockall, and full Rockall in the same endoscopy-confirmed ED cohort and evaluated their associations with 4 clinically relevant outcomes.

## 2. Methods

### 2.1. Study design and setting

This retrospective observational study was conducted at a tertiary academic center between January 1, 2016 and 1 June 1, 2019.

### 2.2. Participants

Adults aged 18 years or older were eligible if they presented with suspected upper gastrointestinal bleeding (UGIB) (hematemesis, coffee-ground emesis, melena, and/or hematochezia) and an UGIB source was confirmed by endoscopy in the ED pathway. During the study period, 665 ED presentations were screened, and 388 patients underwent upper endoscopy within the relevant care pathway. Patients were excluded if endoscopy was not performed, if endoscopy had been performed at an outside center before transfer, if the medical record was inaccessible or materially incomplete for score calculation, or if the presentation involved trauma or pregnancy. After exclusion of 21 inaccessible records, 15 records with insufficient data for score calculation, and 5 pregnancy-related presentations, 347 patients comprised the analytic cohort. Accordingly, the study represents a selected endoscopy-confirmed subgroup rather than an unselected ED presentation cohort with suspected UGIB.

### 2.3. Variables and outcomes

The dataset included age, sex, vital signs, blood urea nitrogen, hemoglobin, international normalized ratio, albumin, Glasgow Coma Scale score, syncope at presentation, symptoms of melena and hematemesis, selected comorbidities, endoscopic findings, endoscopic therapy, transfusion requirement, rebleeding, and index-hospital disposition.

During the index hospitalization, patients were categorized as discharged directly after endoscopy, discharged after hospitalization, or deceased during the same hospitalization. Thirty-day mortality was analyzed separately and was ascertained from hospital records and national death records.

Rebleeding was defined as recurrent hematemesis and/or melena, or hemodynamic instability with rebleeding confirmed on repeat endoscopy, within 30 days of the index presentation. Outcome ascertainment was based on hospital records accessed through the national health record system.

### 2.4. Risk scores

For each patient, AIMS65, GBS, pre-Baylor, full Baylor, pre-Rockall, and full Rockall were calculated using the variables available in the study records. Pre-endoscopic scores were based on pre-endoscopic variables, whereas full Baylor and full Rockall incorporated endoscopic findings.

### 2.5. Statistical analysis

Statistical analyses were performed using SPSS (IBM Corp.), and MedCalc (MedCalc Software Ltd.) was used for diagnostic-performance analyses. Continuous variables were summarized as mean ± standard deviation, median, and minimum–maximum values, whereas categorical variables were summarized as counts and percentages. Distributional assessment was based on normality testing and skewness–kurtosis evaluation. Because the variables were not normally distributed, Kruskal–Wallis, Mann–Whitney *U*, chi-square, and Spearman correlation tests were applied, as appropriate.

Receiver operating characteristic (ROC) analyses were performed to evaluate 30-day mortality, transfusion requirement, and rebleeding across all 6 scores. For endoscopic therapy, ROC analysis was performed for AIMS65, GBS, pre-Baylor, and pre-Rockall. Discrimination was summarized by area under the curve (AUC) with 95% confidence intervals (CIs). For statistically significant ROC models, sensitivity, specificity, positive and negative likelihood ratios, positive and negative predictive values, and cutoff values were calculated. A 2-sided *P* value <.05 was considered statistically significant.

### 2.6. Ethics

The study was approved by the Institutional Clinical Research Ethics Committee (January 23, 2020; decision no. I1-47-20).

## 3. Results

Of 665 ED presentations screened during the study period, 388 underwent upper endoscopy and 347 patients met the analytic criteria after exclusion of 21 inaccessible records, 15 records with missing data required for score calculation, and 5 pregnancies. The patient-selection process is summarized in Figure 1.

**Figure 1.**
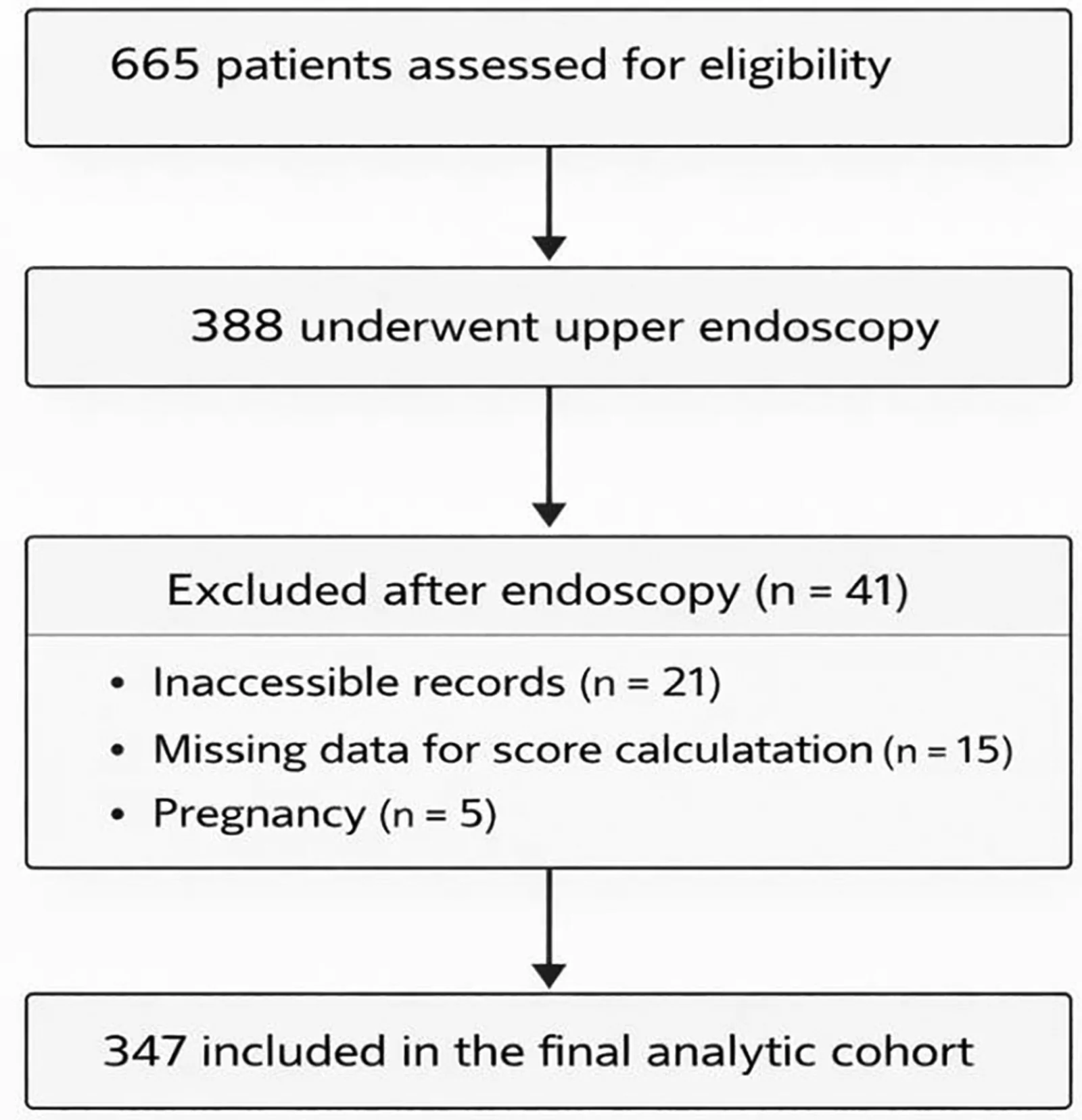
Study flow diagram.

Baseline demographic, presentation, and laboratory characteristics of the analytic cohort are summarized in Table 1.

**Table 1:** Baseline demographic, presentation, and laboratory characteristics (n = 347).

Variable	Value
Age, yr	65.54 ± 15.82
Male sex	217 (62.6%)
Systolic blood pressure, mm Hg	114.5 ± 19.6
Diastolic blood pressure, mm Hg	71.6 ± 13.0
Pulse, beats/min	89.1 ± 20.0
Blood urea nitrogen, mg/dL	39.21 ± 27.39
Hemoglobin, g/dL	9.50 ± 2.99
INR	1.92 ± 2.80
Albumin, g/dL	3.34 ± 0.72
Glasgow Coma Scale <15	30 (8.6%)
Syncope at presentation	6 (1.7%)
Melena	237 (68.3%)
Hematemesis	135 (38.9%)

Data are presented as mean ± standard deviation or n (%).

INR = international normalized ratio.

Comorbidities, endoscopic findings, and clinically relevant outcomes are summarized in Table 2. Kidney failure was the most frequent recorded comorbidity (61.1%), followed by major additional comorbidity (52.2%) and ischemic heart disease (40.3%). Index-hospital disposition comprised direct discharge after endoscopy in 27 patients (7.8%), discharge after hospitalization in 282 (81.3%), and in-hospital death in 38 (11.0%). The separate 30-day mortality endpoint used in ROC analyses comprised 46 deaths (13.3%). Blood transfusion occurred in 205 patients (59.1%), endoscopic therapy in 29 (8.4%), and rebleeding in 71 (20.5%).

**Table 2:** Comorbidities, endoscopic findings, and clinically relevant outcomes.

Variable	Value
Kidney failure	212 (61.1%)
Major additional comorbidity	181 (52.2%)
Ischemic heart disease	140 (40.3%)
Malignancy	108 (31.1%)
Heart failure	91 (26.2%)
Liver failure	91 (26.2%)
Liver disease	68 (19.6%)
Direct discharge after endoscopy	27 (7.8%)
Discharge after hospitalization	282 (81.3%)
In-hospital death	38 (11.0%)
30-d mortality events used in ROC analysis	46 (13.3%)
Upper GI malignancy on endoscopy	34 (9.8%)
Nonmalignant endoscopic diagnosis	120 (34.6%)
No identifiable lesion on endoscopy	193 (55.6%)
No active bleeding stigma	292 (84.1%)
Clot	28 (8.1%)
Visible vessel	3 (0.9%)
Active bleeding	24 (6.9%)
Blood transfusion	205 (59.1%)
Endoscopic therapy	29 (8.4%)
Both endoscopic therapy and transfusion	21 (6.1%)
Rebleeding	71 (20.5%)

Data are presented as n (%).

GI = gastrointestinal, ROC = receiver operating characteristic.

For descriptive context, score distributions according to index-hospital disposition are summarized in Table 3. All 6 scores differed significantly across disposition groups, with the highest mean values observed among patients who died during the index hospitalization and the lowest among those discharged directly after endoscopy.

**Table 3:** Descriptive score distribution according to index-hospital disposition.

Score	Overall mean ± SD	In-hospital death mean ± SD	Discharge after hospitalization mean ± SD	Direct discharge after endoscopy mean ± SD	*P*
AIMS65	1.36 ± 1.05	2.16 ± 1.15	1.30 ± 1.01	0.78 ± 0.70	<.001
Pre-Baylor	10.90 ± 3.35	11.97 ± 2.47	11.03 ± 3.25	8.11 ± 4.05	<.001
Full Baylor	11.41 ± 3.63	12.68 ± 2.52	11.51 ± 3.56	8.48 ± 4.26	<.001
GBS	9.52 ± 4.34	10.97 ± 2.95	9.59 ± 6.78	6.78 ± 4.12	<.001
Pre-Rockall	4.41 ± 2.42	5.82 ± 1.67	4.37 ± 2.81	2.81 ± 2.42	<.001
Full Rockall	5.23 ± 2.68	6.92 ± 2.02	5.18 ± 3.41	3.41 ± 2.79	<.001

Values are presented as mean ± standard deviation.

AIMS65 = albumin, international normalized ratio, mental status, systolic blood pressure, age ≥65 years, GBS = Glasgow-Blatchford score, SD = standard deviation.

Thirty-day mortality discrimination is shown in Table 4. AIMS65 showed the highest AUC (0.724, 95% CI 0.647–0.802). Full Rockall and pre-Rockall also showed statistically significant but more modest discrimination, whereas full Baylor did not reach statistical significance. Threshold-based diagnostic metrics for the statistically significant mortality ROC models are provided in Table S1, Supplemental Digital Content 1.

**Table 4:** Discriminatory performance of risk scores for 30-day mortality.

Score	AUC (95% CI)	*P*	Best cutoff	Sensitivity (%)	Specificity (%)
AIMS65	0.724 (0.647–0.802)	<.001	>1	69.57	63.79
GBS	0.607 (0.520–0.694)	.019	>6	89.13	26.58
Pre-Baylor	0.635 (0.550–0.719)	.003	>12	52.17	64.45
Full Baylor	0.562 (0.484–0.639)	.179	–	–	–
Pre-Rockall	0.656 (0.585–0.728)	.001	>3	89.13	39.87
Full Rockall	0.676 (0.604–0.749)	<.001	>3	97.83	29.90

AIMS65 = albumin, international normalized ratio, mental status, systolic blood pressure, age ≥65 years, AUC = area under the curve, CI = confidence interval, GBS = Glasgow-Blatchford score.

All 6 scoring systems were significantly associated with transfusion requirement (Table 5). GBS showed the highest AUC (0.784, 95% CI 0.734–0.834) and the best apparent discrimination in this cohort. The remaining systems showed lower but statistically significant AUCs. Threshold-based diagnostic metrics for transfusion prediction are provided in Table S2, Supplemental Digital Content 2.

**Table 5:** Discriminatory performance of risk scores for blood transfusion requirement.

Score	AUC (95% CI)	*P*	Best cutoff	Sensitivity (%)	Specificity (%)
AIMS65	0.645 (0.586–0.703)	<.001	>1	49.76	72.54
GBS	0.784 (0.734–0.834)	<.001	>8	77.56	71.13
Pre-Baylor	0.611 (0.550–0.671)	<.001	>10	72.20	47.89
Full Baylor	0.614 (0.554–0.674)	<.001	>11	62.93	57.75
Pre-Rockall	0.649 (0.591–0.707)	<.001	>4	62.93	62.68
Full Rockall	0.656 (0.599–0.714)	<.001	>6	43.90	79.58

AIMS65 = albumin, international normalized ratio, mental status, systolic blood pressure, age ≥65 years, AUC = area under the curve, CI = confidence interval, GBS = Glasgow-Blatchford score.

For endoscopic therapy, only GBS reached nominal statistical significance in ROC analysis (Table 6). However, discrimination was weak (AUC 0.604, 95% CI 0.551–0.656), and the observed threshold performance should be interpreted cautiously. The remaining pre-endoscopic scores did not show statistically significant discrimination for this endpoint. Threshold-based diagnostic metrics for the statistically significant endoscopic-therapy ROC model are provided in Table S3, Supplemental Digital Content 3.

**Table 6:** Discriminatory performance of pre-endoscopic risk scores for endoscopic therapy.

Score	AUC (95% CI)	*P*	Best cutoff	Sensitivity (%)	Specificity (%)
AIMS65	0.527 (0.473–0.580)	.597	–	–	–
GBS	0.604 (0.551–0.656)	.047	>12	44.83	74.21
Pre-Baylor	0.517 (0.463–0.571)	.748	–	–	–
Pre-Rockall	0.520 (0.466–0.573)	.703	–	–	–

Endoscopic therapy was analyzed only for the pre-endoscopic scores evaluated in the study analysis (AIMS65, GBS, pre-Baylor, and pre-Rockall).

AIMS65 = albumin, international normalized ratio, mental status, systolic blood pressure, age ≥65 years, AUC = area under the curve, CI = confidence interval, GBS = Glasgow-Blatchford score.

Rebleeding discrimination is presented in Table 7. GBS showed the highest AUC (0.703, 95% CI 0.639–0.767). AIMS65, full Baylor, pre-Rockall, and full Rockall also reached statistical significance but with more modest discrimination, whereas pre-Baylor did not. Threshold-based diagnostic metrics for statistically significant rebleeding ROC models are provided in Table S4, Supplemental Digital Content 4.

**Table 7:** Discriminatory performance of risk scores for rebleeding.

Score	AUC (95% CI)	*P*	Best cutoff	Sensitivity (%)	Specificity (%)
AIMS65	0.650 (0.579–0.720)	<.001	>1	59.15	64.13
GBS	0.703 (0.639–0.767)	<.001	>9	84.51	54.71
Pre-Baylor	0.564 (0.492–0.637)	.094	-	-	-
Full Baylor	0.593 (0.520–0.666)	.016	>11	67.61	48.91
Pre-Rockall	0.637 (0.569–0.706)	<.001	>4	73.24	52.90
Full Rockall	0.671 (0.601–0.740)	<.001	>5	73.24	55.80

AIMS65 = albumin, international normalized ratio, mental status, systolic blood pressure, age ≥65 years, AUC = area under the curve, CI = confidence interval, GBS = Glasgow-Blatchford score.

All scoring systems were positively correlated with 1 another (Table 8). The strongest correlations were observed between pre-Baylor and full Baylor (*r* = 0.905) and between pre-Rockall and full Rockall (*r* = 0.931), whereas the remaining pairwise correlations were of moderate magnitude.

**Table 8:** Correlation matrix of the 6 upper gastrointestinal bleeding risk scores.

Score	AIMS65	Pre-Baylor	Full Baylor	GBS	Pre-Rockall	Full Rockall
AIMS65	–					
Pre-Baylor	0.560*	–				
Full Baylor	0.570*	0.905*	–			
GBS	0.496*	0.343*	0.333*	–		
Pre-Rockall	0.540*	0.514*	0.501*	0.535*	–	
Full Rockall	0.498*	0.417*	0.534*	0.486*	0.931*	–

All reported coefficients were positive and statistically significant.

AIMS65 = albumin, international normalized ratio, mental status, systolic blood pressure, age ≥65 years, GBS = Glasgow-Blatchford score.

* Correlation is statistically significant (2-tailed *P* < .001).

## 4. Discussion

The main finding of this study is that score performance was outcome-specific rather than uniform across all endpoints. In this selected ED cohort undergoing upper endoscopy, AIMS65 showed the highest observed discrimination for 30-day mortality, whereas GBS showed the highest observed discrimination for transfusion requirement and rebleeding. For endoscopic therapy, only GBS reached statistical significance, but the corresponding AUC remained low, indicating limited clinical usefulness for predicting the need for endoscopic intervention.

A key strength of this study is the head-to-head evaluation of 6 established UGIB risk scores in the same ED cohort, including both pre-endoscopic and post-endoscopic models. Unlike studies focusing on a single endpoint, we examined 4 clinically relevant outcomes – 30-day mortality, transfusion requirement, endoscopic therapy, and rebleeding – which allowed a more granular assessment of outcome-specific score performance. These findings suggest that no single score can be assumed to perform optimally across all decision points in UGIB care.

The mortality signal observed for AIMS65 is consistent with prior comparative studies, although the magnitude of discrimination in our cohort was more modest. In the international multicenter prospective study by Stanley et al, AIMS65 showed better discrimination for 30-day mortality than GBS.^[10]^ Hyett et al similarly reported superior performance of AIMS65 over GBS for in-hospital mortality,^[11]^ while Robertson et al found AIMS65 to perform at least comparably with the other commonly used scores for mortality prediction.^[12]^ In an ED cohort using the same 30-day mortality endpoint as our study, Tang et al also identified AIMS65 among the best-performing scores.^[14]^ These findings are consistent with the relative advantage of AIMS65 observed in our cohort, although its AUC of 0.724 indicates only moderate discrimination. Differences in mortality definition, case mix, and patient selection across study populations may partly explain variation in absolute performance. Accordingly, AIMS65 should be interpreted as a structured adjunct for mortality-oriented risk assessment rather than as a stand-alone tool capable of replacing bedside evaluation, hemodynamic reassessment, response to resuscitation, or endoscopic judgment.

GBS showed the highest observed discrimination for both transfusion requirement and rebleeding in our cohort, although its performance differed according to the endpoint. Its relative strength for transfusion is consistent with previous comparative studies, in which Hyett et al and Yaka et al found GBS to outperform AIMS65 for predicting blood transfusion.^[11,17]^ In our cohort, GBS achieved an AUC of 0.784 for transfusion, supporting this overall pattern while indicating more modest discrimination. By contrast, rebleeding has been less reliably predicted across previous studies. Stanley et al reported limited performance of the evaluated scores for this outcome,^[10]^ and Chang et al similarly found that none of the conventional scoring systems satisfactorily predicted rebleeding.^[13]^ Although GBS showed the highest observed AUC for rebleeding in our cohort (0.703), this represents only moderate discrimination and should therefore not be interpreted as evidence of reliable standalone prediction.

The endoscopic-therapy endpoint deserves particular restraint in interpretation. Although GBS was the only pre-endoscopic score that reached statistical significance in our cohort, its AUC of 0.604 indicates poor discrimination. In the international multicenter study by Stanley et al, GBS showed substantially better discrimination for endoscopic treatment (AUC 0.75) than AIMS65 (0.62) and admission Rockall (0.61).^[10]^ Pang et al, in a prospective validation study of 1087 patients, similarly reported an AUC of 0.72 for GBS in predicting the need for therapeutic endoscopy, whereas the pre-Rockall score was not useful for this endpoint.^[15]^ More recently, Arikoglu et al reported a different pattern in an emergency-unit cohort: Rockall was the only score with statistically significant discrimination for endoscopic intervention (AUC 0.614), whereas GBS had an AUC of 0.521.^[19]^. These differences suggest that prediction of endoscopic therapy may be particularly sensitive to differences in patient selection, endpoint definition, score timing, and local endoscopic practice. In practical terms, the weak discrimination observed in our study does not support the use of any of the evaluated pre-endoscopic scores as an isolated determinant of endoscopic-treatment decisions.

Compared with the recent emergency-unit study by Arikoglu et al,^[19]^ our findings showed both similarities and differences across individual outcomes. In both studies, GBS showed the strongest discrimination for transfusion requirement, although performance was somewhat lower in our cohort (AUC 0.784 vs. 0.846). Mortality findings were also broadly comparable, with AIMS65 among the better-performing scores in both cohorts, although the relative ranking of the remaining scores differed. In contrast, prediction of endoscopic intervention remained weak in both studies, but the statistically significant score differed: Rockall in the study by Arikoglu et al and GBS in our cohort. These differences may reflect variation in patient selection, endpoint definitions, score sets, and timing of assessment, reinforcing that score performance is dependent on both the clinical context and the outcome being evaluated.^[19,20]^

The post-endoscopic models in our study did not uniformly outperform the pre-endoscopic tools. Earlier Baylor and Rockall literature supports a role for endoscopic information in mortality and rebleeding stratification, but the incremental benefit of these models has not been consistent across all outcomes or all study populations.^[6–8,12,20]^ In our cohort, full Rockall and full Baylor were informative in some analyses, but they did not eliminate the need for lesion-level interpretation, repeated hemodynamic assessment, and overall clinical judgment.

The empirical thresholds identified in this study should be interpreted as cohort-specific findings rather than portable clinical cut-points. For example, the GBS thresholds associated here with transfusion or rebleeding were substantially higher than the low-risk discharge thresholds commonly discussed in guidelines, validation studies, and meta-analyses.^[1–5,18,21]^ This is not contradictory. Rather, it reflects a different target outcome, a selected cohort undergoing endoscopy, and a case mix enriched for clinically important bleeding.

## 5. Limitations

This study has several limitations. First, its retrospective single-center design limits generalizability. Second, because the analytic cohort included only patients who underwent endoscopy within the ED pathway, the findings reflect a selected subgroup rather than all patients presenting to the ED with suspected UGIB. This introduces workup and verification bias, particularly when interpreting the performance of pre-endoscopic scores. Third, the cohort had a substantial comorbidity burden, which may have influenced absolute score distributions and may differ from other ED populations.

Fourth, index-hospital disposition and 30-day mortality were analyzed as separate outcomes. Although this distinction was made deliberately, it should be kept in mind when interpreting descriptive versus predictive analyses. Fifth, endoscopic-therapy discrimination was weak, and the statistically significant result observed for GBS should not be interpreted as evidence of strong procedural predictive utility. Finally, threshold-based findings should be regarded as cohort-specific and require external validation before being considered for broader clinical implementation.

Despite these limitations, the study also has important strengths. It provides a same-cohort comparison of 6 established scoring systems, includes both pre- and post-endoscopic models, and evaluates 4 clinically relevant outcomes while preserving close fidelity to the underlying study dataset.

## 6. Conclusions

In this selected cohort of ED patients with UGIB who underwent endoscopy, no single score performed best across all outcomes. AIMS65 showed the highest observed discrimination for 30-day mortality, whereas GBS performed best for transfusion requirement and rebleeding. By contrast, prediction of endoscopic therapy remained weak across the evaluated pre-endoscopic models. Overall, these findings support an outcome-specific approach to UGIB risk stratification, in which score choice is guided by the clinical endpoint and timing of use rather than by the assumption that 1 tool can reliably serve all purposes.

## Author contributions

**Conceptualization:** Emre Oner.

**Data curation:** Niyazi Ilhan, Yasar Catal, Sinan Genc.

**Formal analysis:** Niyazi Ilhan, Yasar Catal, Sinan Genc.

**Investigation:** Emre Oner, Niyazi Ilhan, Yasar Catal, Sinan Genc.

**Methodology:** Emre Oner, Niyazi Ilhan, Sinan Genc.

**Project administration:** Emre Oner, Niyazi Ilhan, Ayca Koca, Muge Gunalp.

**Resources:** Emre Oner, Yasar Catal, Sinan Genc.

**Supervision:** Ayca Koca, Muge Gunalp.

**Validation:** Ayca Koca, Muge Gunalp.

**Visualization:** Ayca Koca, Muge Gunalp.

**Writing – original draft:** Emre Oner.

**Writing – review & editing:** Emre Oner, Ayca Koca, Muge Gunalp, Sinan Genc.








