# Supplementary material for: Comparative performance of pre- and post-endoscopic risk scores for outcome prediction in endoscopy-confirmed upper gastrointestinal bleeding in the emergency department: A retrospective single-center study
Source: Medicine (Baltimore). 2026 Oct 23;105(43):e51268. doi: 10.1097/MD.0000000000051268 (PMC13706388; doi:10.1097/MD.0000000000051268)
Supplement: Supplementary file 1 [file medi-105-e51268-s001.pdf]

Supplementary Appendix

The tables below summarize threshold-based diagnostic metrics for the statistically significant ROC models identified in the study analyses. Where available, 95% confidence intervals are also provided. Only statistically significant ROC models are presented in these supplementary threshold tables.

Supplementary Table S1. Threshold metrics for 30-day mortality

Score	Cut-off	Sensitivity, % (95% CI)	Specificity, % (95% CI)	+LR (95% CI)	-LR (95% CI)	PPV, % (95% CI)	NPV, % (95% CI)
AIMS65	>1	69.57 (54.2-82.3)	63.79 (58.1-69.2)	1.92 (1.5-2.4)	0.48 (0.3-0.7)	22.7 (18.7-27.2)	93.2 (89.8-95.5)
GBS	>6	89.13 (76.4-96.4)	26.58 (21.7-31.9)	1.21 (1.1-1.4)	0.41 (0.2-1.0)	15.6 (14.1-17.3)	94.1 (87.3-97.4)
Pre-Baylor	>12	52.17 (36.9-67.1)	64.45 (58.8-69.9)	1.47 (1.1-2.0)	0.74 (0.5-1.0)	18.3 (14.1-23.5)	89.8 (86.6-92.3)
Pre-Rockall	>3	89.13 (76.4-96.4)	39.87 (34.3-45.6)	1.48 (1.3-1.7)	0.27 (0.1-0.6)	18.5 (16.5-20.6)	96.0 (91.2-98.2)
Full Rockall	>3	97.83 (88.5-99.9)	29.90 (24.8-35.4)	1.40 (1.3-1.5)	0.07 (0.01-0.5)	17.6 (16.4-18.9)	98.9 (92.8-99.8)

Abbreviations: CI, confidence interval; LR, likelihood ratio; PPV, positive predictive value; NPV, negative predictive value.
