# Supplementary material for: Comparative performance of pre- and post-endoscopic risk scores for outcome prediction in endoscopy-confirmed upper gastrointestinal bleeding in the emergency department: A retrospective single-center study
Source: Medicine (Baltimore). 2026 Oct 23;105(43):e51268. doi: 10.1097/MD.0000000000051268 (PMC13706388; doi:10.1097/MD.0000000000051268)
Supplement: Supplementary file 2 [file medi-105-e51268-s002.pdf]

Supplementary Table S2. Threshold metrics for blood transfusion requirement

Score	Cut-off	Sensitivity, % (95% CI)	Specificity, % (95% CI)	+LR (95% CI)	-LR (95% CI)	PPV, % (95% CI)	NPV, % (95% CI)
AIMS65	>1	49.76 (42.7-56.8)	72.54 (64.4-79.7)	1.81 (1.3-2.4)	0.69 (0.6-0.8)	72.3 (65.9-77.9)	50.0 (45.8-54.2)
GBS	>8	77.56 (71.2-83.1)	71.13 (62.9-78.4)	2.69 (2.1-3.5)	0.32 (0.2-0.4)	79.5 (74.8-83.5)	68.7 (62.5-74.3)
Pre-Baylor	>10	72.20 (65.5-78.2)	47.89 (39.4-56.4)	1.39 (1.2-1.7)	0.58 (0.4-0.8)	66.7 (62.6-70.5)	54.4 (47.4-61.2)
Full Baylor	>11	62.93 (55.9-69.6)	57.75 (49.2-66.0)	1.49 (1.2-1.9)	0.64 (0.5-0.8)	68.3 (63.3-72.8)	51.9 (46.2-57.5)
Pre-Rockall	>4	62.93 (55.9-69.6)	62.68 (54.2-70.6)	1.69 (1.3-2.1)	0.59 (0.5-0.7)	70.9 (65.7-75.5)	53.9 (48.5-59.3)
Full Rockall	>6	43.90 (37.0-51.0)	79.58 (72.0-85.9)	2.15 (1.5-3.1)	0.70 (0.6-0.8)	75.6 (68.4-81.6)	49.6 (45.9-53.2)

Abbreviations: CI, confidence interval; LR, likelihood ratio; PPV, positive predictive value; NPV, negative predictive value.
