# Supplementary material for: Comparative performance of pre- and post-endoscopic risk scores for outcome prediction in endoscopy-confirmed upper gastrointestinal bleeding in the emergency department: A retrospective single-center study
Source: Medicine (Baltimore). 2026 Oct 23;105(43):e51268. doi: 10.1097/MD.0000000000051268 (PMC13706388; doi:10.1097/MD.0000000000051268)
Supplement: Supplementary file 3 [file medi-105-e51268-s003.pdf]

Supplementary Table S3. Threshold metrics for endoscopic therapy

Score	Cut-off	Sensitivity, % (95% CI)	Specificity, % (95% CI)	+LR (95% CI)	-LR (95% CI)	PPV, % (95% CI)	NPV, % (95% CI)
GBS	>12	44.83 (26.4-64.3)	74.21 (69.0-78.9)	1.74 (1.1-2.7)	0.74 (0.5-1.0)	13.7 (9.2-19.8)	93.7 (91.3-95.4)

Abbreviations: CI, confidence interval; LR, likelihood ratio; PPV, positive predictive value; NPV, negative predictive value.
