# Supplementary material for: Comparative performance of pre- and post-endoscopic risk scores for outcome prediction in endoscopy-confirmed upper gastrointestinal bleeding in the emergency department: A retrospective single-center study
Source: Medicine (Baltimore). 2026 Oct 23;105(43):e51268. doi: 10.1097/MD.0000000000051268 (PMC13706388; doi:10.1097/MD.0000000000051268)
Supplement: Supplementary file 4 [file medi-105-e51268-s004.pdf]

Supplementary Table S4. Threshold metrics for rebleeding

Score	Cut-off	Sensitivity, % (95% CI)	Specificity, % (95% CI)	+LR (95% CI)	-LR (95% CI)	PPV, % (95% CI)	NPV, % (95% CI)
AIMS65	>1	59.15 (46.8-70.7)	64.13 (58.2-69.8)	1.65 (1.3-2.1)	0.64 (0.5-0.9)	29.8 (24.8-35.3)	85.9 (82.0-89.1)
GBS	>9	84.51 (74.0-92.0)	54.71 (48.6-60.7)	1.87 (1.6-2.2)	0.28 (0.2-0.5)	32.4 (29.0-36.1)	93.2 (88.8-96.0)
Full Baylor	>11	67.61 (55.5-78.2)	48.91 (42.9-55.0)	1.32 (1.1-1.6)	0.66 (0.5-0.9)	25.4 (21.8-29.3)	85.4 (80.4-89.3)
Pre-Rockall	>4	73.24 (61.4-83.1)	52.90 (46.8-58.9)	1.55 (1.3-1.9)	0.51 (0.3-0.8)	28.6 (24.9-32.6)	88.5 (83.7-92.0)
Full Rockall	>5	73.24 (61.4-83.1)	55.80 (49.7-61.7)	1.66 (1.4-2.0)	0.48 (0.3-0.7)	29.9 (26.0-34.1)	89.0 (84.5-92.4)

Abbreviations: CI, confidence interval; LR, likelihood ratio; PPV, positive predictive value; NPV, negative predictive value.
